# Going Native? Yes, If Allowed by Cross-Linguistic Similarity

**DOI:** 10.3389/fpsyg.2021.742127

**Published:** 2021-11-10

**Authors:** Gillen Martínez de la Hidalga, Adam Zawiszewski, Itziar Laka

**Affiliations:** Department of Linguistics and Basque Studies, Faculty of Arts, University of the Basque Country, Vitoria-Gasteiz, Spain

**Keywords:** non-native language processing, event-relate potentials, unergative vs. unaccusative predicates, subject-verb agreement, phi-features, bilingualism

## Abstract

Can native competence be achieved in a second language? Here, we focus on the Language Distance Hypothesis that claims that early and proficient bilinguals can achieve native competence for grammatical properties shared by their two languages, whereas unshared grammatical properties pose a challenge for native-like syntactic processing. We present a novel behavioral and Event-Related Potential (ERP) study where early and proficient bilinguals behave native-like in their second language when processing (a) argument structure alternations in intransitive sentences involving agent vs. patient subjects and (b) subject verb agreement, both of which are grammatical properties shared by their two languages of these bilinguals. Compared to native Basque bilinguals (L2Spanish) on the same tasks, non-natives elicited similar sentence processing measures: (a) in the acceptability task they reacted faster and more accurately to unaccusative sentences than to unergatives and to person than number violations: (b) they generated a larger P600 for agreement violations in unaccusative sentences than unergatives; (c) they generated larger negativity and positivity effects for person than for number violations. Previous studies on Basque-Spanish bilinguals find that early and proficient non-natives display effects distinct from natives in both languages when processing grammatical properties where Basque and Spanish diverge, such as argument alignment (ergative/nominative) or word order type (OV/VO), but they perform native-like for shared properties such as subject agreement and word meaning. We contend that language distance, that is, the degree of similarity of the languages of the bilingual is a crucial factor that deserves further and detailed attention to advance our understanding of when and how bilinguals can *go native* in a second language.

## Introduction

Can non-native speakers attain native-like competence in grammatical processing? Research carried out throughout the last decades has identified key factors to take into account when studying non-native syntactic processing, namely age of acquisition (AoA), proficiency, similarity between L1 and L2 and active use of the language (Caffarra et al., 2015; Hartshorne et al., 2018; Brice et al., 2019).

In second language acquisition, syntax is reported to be harder to acquire than other aspects of language (Ojima et al., 2005; Kotz, 2009; Vandenberghe et al., 2019). It has also been shown that AoA and the level of proficiency play a big role in attaining native-like performance (i.e., Weber-Fox and Neville, 1996; Wartenburger et al., 2003). Weber-Fox and Neville (1996), for instance, used ERPs to test Chinese-English adult bilinguals exposed to English at different age during the life span (1–3, 4–6, 7–10, 11–13, and after 16 years of age) and asked the participants to read sentences containing syntactic and semantic anomalies. Results revealed significant AoA effects for syntactic processing (phrase structure, specificity and subjacency constraints), that is, in comparison to English monolinguals, behavioral and electrophysiological measures of Chinese-English bilinguals were affected by a delay in L2 exposure as short as 1–3 years. By contrast, regarding semantic anomalies, only subjects exposed to English after 11–13 years showed differences as compared to natives. Wartenburger et al. (2003), in turn, used the fMRI method to test the effects of AoA and proficiency in three groups of Italian-German bilinguals who learned their L2 at different ages and had different proficiency levels (early AoA (= at birth), high proficiency group; late AoA (>6 years), high proficiency group and late AoA (>6 years), low proficiency group). Participants read sentences in their L1 and L2 containing syntactic (gender, number or case disagreement) and semantic anomalies (i.e., “The deer shoots the hunter”). Results revealed that differences in the fMRI pattern reported for the syntactic task were due to the delay in AoA, while the pattern of brain activity for semantic judgment depended on the level of proficiency, supporting the findings of Weber-Fox and Neville (1996).

On the other hand, several studies affirm that high proficiency L2 speakers can attain native-like performance (Friederici et al., 2002; Rossi et al., 2006; Hernandez et al., 2007; Kotz et al., 2008) regardless of their late AoA, thus challenging the Critical Period Hypothesis (Lenneberg, 1967). Friederici et al. (2002) used ERP measures to test adult German learners (AoA = 24.1 years) of an artificial language and showed a similar ERP pattern to that reported for native speakers of German on a similar task in natural language. According to the authors, these results indicate that a language learned late can be processed in a native-like way. Similarly, Rossi et al. (2006) showed that high-proficiency late L2 Italian-German and German-Italian learners (AoA > 10 years) display the same ERP components as native speakers when processing word category and subject-verb agreement syntactic violations, suggesting that with a high proficiency L2 learners can show native-like responses regardless of the late AoA.

Finally, some studies report differences between native and non-natives regarding certain syntactic phenomena but not others (Zawiszewski et al., 2011; Foucart and Frenck-Mestre, 2012; Erdocia et al., 2014; Díaz et al., 2016; Zawiszewski and Laka, 2020). Zawiszewski et al. (2011), Díaz et al. (2016), and Zawiszewski and Laka (2020) examined the processing of case morphology and Erdocia et al. (2014) the processing of word order comparing native and non-native speakers of Basque (L1Spanish) and found that non-natives, despite an early AoA and high competence in their L2 did not process these two aspects of Basque grammar like natives. Since case alignment and basic word order are two main grammatical features that Basque and Spanish do not share (Basque is ergative and OV, Spanish is nominative and VO), they concluded that linguistic distance, that is, the degree of similarity of the bilingual’s grammars was a relevant factor in final attainment in second language processing.

These different sets of findings reported in the L2 processing literature have been accounted by many theoretical proposals. Some posit that L2 acquisition strongly depends on the L1 and thus the results can be interpreted in terms of a positive or negative transfer (i.e., The Unified Model of Language Acquisition, Hernandez et al., 2005; MacWhinney, 2005). Schwartz and Sprouse (1996) also suggest that L2 acquisition hinges on the features available in the L1 (the Full Transfer/Full Access model) and this view is also compatible with the Interpretability Hypothesis (Tsimpli and Dimitrakopoulou, 2007), which posits that only interpretable features are accessible to the L2 learners while the uninterpretable ones are subject to critical period constraints and, consequently, inaccessible to L2 learners. The possibility of syntactic information being shared between both languages has been also suggested by Hartsuiker et al. (2004) (Shared Syntax Account): it suggests that grammatical rules that are the same in L1 and L2 are represented once. In other words, L2 learners would rely on their L1 whenever using a grammatical structure present in the two languages (see also Zawiszewski and Laka, 2020, for similar assumptions), Conversely, Clahsen and Felser (2006) put forward the Shallow Structure Hypothesis and suggest that late L2 learners are not able to process syntax in a native-like way and have to rely to a large extent on semantic/pragmatic information (see also Steinhauer et al., 2009 for a discussion).

## The Present Study

The present study sought to examine the processing of intransitive predicates in Basque by early and proficient L1Spanish—L2Basque bilinguals. To this purpose, we used grammatical and ungrammatical person and number agreement manipulations and compared the results to those previously reported by Martinez de la Hidalga et al. (2019) for natives.

The distinction between intransitives whose sole argument is an agent (unergatives) and intransitives whose sole argument is a theme (unaccusatives) is a general property of grammars [Unaccusative Hypothesis (UH), Perlmutter, 1978], and both Basque and Spanish differentiate these two types of predicates. More precisely, the UH claims that unaccusative involve more complex derivations than unergatives because themes are promoted to subjects or undergo movement and leave a trace (Burzio, 1986), whereas agents are born as subjects. Importantly, some authors propose for the unaccusative verbs in Basque the same derivation as stated by the UH (Ortiz de Urbina, 1989), while others claim no need for the extra derivational step (Laka, 2006a, b; Levin, 1983). More complex derivations are usually related to a greater processing cost (longer reading or reaction times, larger ERP signatures) as compared to less complex structures (i.e., Matzke et al., 2002). Consequently, larger processing cost is expected for unaccusatives in comparison to unergatives. Subject agreement is also a shared property between Basque and Spanish, and both grammars represent it by means of person and number features. Unaccusative verbs have been found to be harder to learn than unergatives for second language learners at initial stages (Yuan, 1999; Oshita, 2001; Montrul, 2005; *inter alia*). Oshita (2001) for instance, put forth the Unaccusative Trap Hypothesis (UTH), arguing that L2 learners assume at first all intransitive predicates to be unergatives. As proficiency increases, however, learners notice that unaccusatives function differently and start making differences between the two predicates, and at higher levels of proficiency they are found to perform native-like regarding this linguistic dimension.

In a previous study carried out in Basque, Martinez de la Hidalga et al. (2019) investigated Basque-Spanish bilinguals in order to test the UH hypothesis and phi-feature processing. Results revealed that in the acceptability task the participant reacted faster and more accurately to unaccusative sentences than to unergatives and to person than number violations and they generated a larger P600 for agreement violations in unaccusative sentences than in unergatives. Furthermore, they generated larger negativity and positivity effects for person than for number violations. Overall, the results revealed greater processing costs for unergatives than for unaccusatives and the authors interpreted these findings as evidence providing support for different structural representations of both types of predicates. However, the prediction of higher processing cost for unaccusatives than for unergatives was not confirmed, supporting the idea of an inherent rather than structural nature of case in Basque (Levin, 1983; Laka, 2006a, b). Regarding agreement features, native speakers processed person and number features separately, the person being far more salient than the number (see Carminati, 2005; Zawiszewski et al., 2016; Mancini, 2018, for more information on the processing of person and number features).

### Hypotheses and Predictions

Our working hypothesis is the Language Distance Hypothesis (LDH, after Zawiszewski and Laka, 2020): no differences are expected for processing traits of L2 that are present in L1, whereas even at an early AoA and high proficiency in L2, native vs. non-native differences will arise in the processing grammatical properties of L2 not present in L1. Previous studies in Basque investigating ergative case morphology (Díaz et al., 2011; Zawiszewski et al., 2011; Zawiszewski and Laka, 2020) and word order processing (Erdocia et al., 2014) in native and early and highly proficient Spanish-Basque bilinguals found differences between both populations, attributed by the authors to the diverging grammatical characteristics of Basque and Spanish.

In the present study, the experimental manipulations involve grammatical traits shared by both Basque and Spanish, namely the distinction between unaccusative vs. unergative predicates, and person vs. number features in subject-verb agreement. However, despite the fact that both Basque and Spanish distinguish between unaccusative and unergative predicates, in Basque agents bear an ergative case marking and themes are morphologically unmarked. In contrast, in Spanish all subjects are morphologically indistinguishable.

We tentatively hypothesize that, given the early AoA and high proficiency of the non-native speakers under study, a similar pattern of results to that reported in Martinez de la Hidalga et al. (2019) will emerge: (a) faster and more accurate responses to unaccusative sentences than to unergative ones and to person violations than number violations in the acceptability task; (b) a general N400–P600 pattern as an ERP response to verb agreement violations, unaccusative violations generated a larger positivity as compared to unergatives and person feature violations generated a larger negativity as compared to number feature violations in the early time window; and (c) person violations in the unergative condition generated a larger positivity as compared to number violations, and larger positivity obtained for number violations in the unaccusative condition as compared to number violations in the unergative condition in the late time window (P600 effect). The predictions made by the LDH are also compatible with the Shared Syntax account (Hartsuiker et al., 2004).

### Participants

In this experiment 26 early and highly proficient non-native speakers of Basque, whose L1 was Spanish^1^ took part in the experiment (five males; mean age 20.5 years, *SD* = 2.67; AoA = 3.31 years, *SD* = 1.3). Data from two participants were excluded as a result of excessive eye movements and other artifacts. All participants were schooled in Basque from early childhood and were therefore highly proficient in Basque (see Table 1 for details) as revealed by the fact that 21 participants had a certified C1 level in Basque and the remaining 3 were completing their undergraduate degree in Basque.

**TABLE 1:** The following seven-point scale was applied for measuring the relative use of language: 1 = I speak only Basque, 2 = I speak mostly Basque, 3 = I speak Basque 75% of the time, 4 = I speak Basque and Spanish with similar frequency, 5 = I speak Spanish 75% of the time, 6 = I speak mostly Spanish, 7 = only Spanish.

Relative use of language		
Before primary school (0–3 years)		6.54 (0.72)
Primary school (4–12 years)		
School		2.88 (1.42)
Home		6.54 (0.72)
Others		5.86 (1.08)
Secondary school (12–18 years)		
School		3.58 (1.07)
Home		6.67 (0.56)
Others		5.92 (0.93)
At time of testing		
University/work		4.5 (1.69)
Home		6.63 (0.58)
Others		5.46 (1.02)

**Self-rated proficiency**	**Basque**	**Spanish**

Speaking	5.92 (0.58)	6.88 (0.34)
Comprehension	6.42 (0.5)	6.92 (0.28)
Reading	6.46 (0.51)	6.83 (0.38)
Writing	6 (0.59)	6.67 (0.48)

*Proficiency level was determined by using the following four-point scale: 7 = native-like proficiency,6 = full proficiency, 5 = working proficiency, 4 = limited proficiency. SDs values are in parentheses.*

## Materials and Methods

416 sentences distributed in four lists (256 experimental and 160 fillers) were created. The materials were organized according to the manipulations used in the experiment (2 × 2 × 2 design): predicate type (unaccusative vs. unergative), feature (person and number), and grammaticality (grammatical and ungrammatical) (see Table 2). For person conditions 2nd person was used in the grammatical condition and for 1st person was used in the ungrammatical manipulation. For number conditions, the design used in Mancini et al. (2011) was followed: 3rd singular vs. plural manipulations. The critical words were the auxiliary verbs, always preceded by the main verbs and followed by three words all verbs were controlled for length and frequency.

**TABLE 2:** Experimental conditions with examples of experimental materials.

Conditions			Sentence examples
Predicate type	Feature	Grammaticality	
Unaccusative	Person	Grammatical	1. Zu gaur goizean bueltatu zara Bilbotik. you-ABS today morning.in returned 2SG.ABS-be Bilbao-from “You have come back from Bilbao this morning.”
		Ungrammatical	2. *Zu gaur goizean bueltatu naiz Bilbotik. you-ABS today morning.in returned 1SG.ABS-be Bilbao-from
	Number	Grammatical	3. Hura gaur goizean bueltatu da Bilbotik. 3.SG-ABS today morning.in returned 3SG.ABS-be Bilbao-from
		Ungrammatical	4. *Hura gaur goizean bueltatu dira Bilbotik. 3.SG-ABS today morning.in returned 3PL.ABS-be Bilbao-from
Unergative	Person	Grammatical	5. Zuk goizean biziki sufritu duzu aurkezpenean. you-ERG morning a.lot suffered have-2SG.ERG presentation-the-at “You have suffered a lot this morning at the presentation.”
		Ungrammatical	6. *Zuk goizean biziki sufritu dut aurkezpenean. you-ERG morning a.lot suffered have-1SG.ERG presentation-the-at
	Number	Grammatical	7. Hark goizean biziki sufritu du aurkezpenean. 3.SG-ERG morning a.lot suffered have-3SG.ERG presentation-the-at
		Ungrammatical	8. *Hark goizean biziki sufritu dute aurkezpenean. 3.SG-ERG morning a.lot suffered have-3PL.ERG presentation-the-at

*stands for ungrammatical sentences.

### Procedure

Personal computers (Windows 7 operating system) and Presentation software (version 16.3) were used to present the stimuli on screen. Before the experiment started, participants were told about the EEG procedure and seated comfortably in a quiet room in front of a 24 inch monitor. The experiment was conducted in the Experimental Linguistics Laboratory at the University of the Basque Country (UPV/EHU) in Vitoria-Gasteiz. Participants conducted an acceptability judgment task, were both accuracy and reaction times were recorded. Sentences were displayed in the middle of the screen word by word for 350 ms (ISI = 250). A fixation cross (+) indicated the beginning of each sentence trial. After each trial the words *zuzen?* “correct?” or *over?* “incorrect?” were displayed in the screen, and participants had to judge the acceptability of the previously shown sentence as either correct or incorrect. Half of participants used the left hand for correct responses (left Ctrl) and the other half the right hand (right Intro).

All sentences were randomly distributed in four blocks. Each block lasted approximately 10 min each and participants had a short break between each block, for as long as they needed. Before the experiment began, participants ran a short training session consisting of three trials. They were instructed to avoid blinking or moving while the sentences were being displayed and to make the acceptability judgment as fast and accurately as possible. The whole experiment, including electrode-cap application and removal, lasted about 1 h 15 m.

### EEG Recording

The EEG was recorded from 32 active electrodes secured in an elastic cap (Acticap System, Brain Products). Electrodes were set on standard positions according to the extended Internationals 10–20 system accordingly: Fp1/Fp2, Fz, F3/F4, F7/F8, FC5/FC6, FC1/FC2, T7/T8, C3/C4, Cz, CP5/CP6, CP1/CP2, P7/P8, P3/P4, Pz, O1/02, Oz, LM, VEOG and HEOG. All recordings were referenced to right mastoid position and re-referenced off-line to the linked mastoids. Vertical and horizontal eye movements and blinks were monitored by means of two electrodes positioned beneath and to the right of the right eye. Electrode impedance was kept below 5 kOhm at all scalp and below 10 kOhm for the eye electrodes. The electrical signals were digitized online at a rate of 500 Hz by a Brain Vision amplifier system and filtered offline within a band pass of 0.1–35 Hz. After the EEG data were recorded, the ocular correction procedure (Gratton et al., 1983) as well as the artifact rejection procedure were applied (offline). Trials with other artifacts with any voltage exceeding 150 μV and voltage steps between two sampling points exceeding 35 μV were removed.

### Data Analysis

For the data analysis four types of subject agreement violations were compared: unaccusative person violations (*zara* “be.2SG” vs. ^∗^*naiz* “be.1SG”; conditions 1 vs. 2 in Table 1, respectively); unaccusative number violations (*da* “be.3SG” vs. ^∗^*dira* “be.3PL”; conditions 3 vs. 4 in Table 1, respectively); unergative person violations (*duzu* “have.2SG” vs. ^∗^*dut* “have.1SG”; conditions 5 vs. 6 in Table 1, respectively); unergative number violations (*du* “have.3SG” vs. ^∗^*dute* “have.3PL”; conditions 7 vs. 8 in Table 1, respectively).

For the ERP measures, segments were created from 200 ms before and 1,000 ms after the onset of the critical words (the auxiliary) in the sentences. The trials associated with each sentence type were averaged for each participant. The EEG 200 ms prior to the onset was also used as a baseline for all sentence type comparisons.

Three hundred to four hundred milliseconds and four hundred to seven hundred milliseconds temporal windows were selected for statistical analysis in all conditions based on the literature and visual inspection of the data. After the stimuli were recorded and averaged, analyses of variance (ANOVA) were carried out in nine regions of interest that were computed out of 27 electrodes: lateral electrodes: left frontal (F7, F3, FC5), left central (T7, FP5, C3), left parietal (P7, P3, O1), right frontal (F4, F8, FC6), right central (C4, FP6, T8), and right parietal (P8, P4, O2); midline electrodes: frontal (Fp1, Fz, Fp2), central (FC1, Cz, FC2), and parietal (CP1, Pz, CP2). Repeated-measures ANOVAs were conducted in all experimental manipulations and trials (correctly and incorrectly judged trials) for each window of time using five within-subjects factors: grammaticality (2 levels: grammatical, ungrammatical), type (2 levels: unaccusative, unergative), feature (2 levels: person, number), hemisphere (2 levels: left, right), and region (3 levels: frontal, central and parietal). Midline (frontal, central, and parietal) electrodes were analyzed independently. Whenever the sphericity of variance was violated (Greenhouse and Geisser, 1959) correction was applied to all the data with greater than one degree of freedom in the numerator. Finally, further statistical comparisons were carried out (split by the grammaticality condition) whenever we found a statistically significant interaction. We only consider effects for the type, feature, hemisphere or region factors when there is an interaction with grammaticality.

For the behavioral results, error rates and response latencies of all the trials repeated measures ANOVAs were performed with grammaticality (two levels: grammatical, ungrammatical), type (two levels: unaccusative, unergative) and feature (two levels: person, number) conditions as within-subject factors. Subsequent comparisons (by subject and by item) were carried out whenever a grammatical interaction was significant.

## Results

### Behavioral Results

**FIGURE 1:**
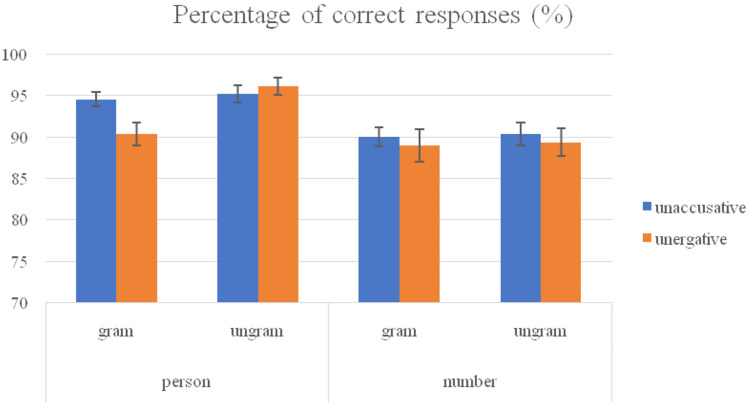
Percentage of correct responses (%) and standard deviation error (SDE) of non-native speakers of Basque.

Here, results concerning the acceptability task and reaction times are presented. Participants were very accurate in the acceptability task (mean accuracy of 91.84%, SDE = 1.3), as was to be expected given their high proficiency in Basque (see Figure 1).

Regarding acceptability judgment errors, the analysis showed a marginally significant GRAMMATICALITY effect in the analysis by item [*F1*(1, 23) = 1.8, *p* = 0.193; *F2*(1, 253) = 3.03, *p* = 0.083] revealing higher accuracy for the ungrammatical sentences as compared to the grammatical ones (92.74% vs. 90.95%). The analysis of accuracy also revealed a main FEATURE effect [*F1*(1, 23) = 23.4, *p* < 0.001; *F2*(1, 253) = 24.62, *p* < 0.001] indicating that participants were more accurate with conditions containing person feature (94.04%) compared to conditions containing number feature (89.65%).

The GRAMMATICALITY^∗^FEATURE interaction turned out to be statistically significant as well [*F1*(1, 23) = 5.34, *p* = 0.03; *F2*(1, 253) = 3.22, *p* = 0.074]. The analyses by grammaticality factor showed that participants were significantly less accurate with grammatical person (92.45%) than with ungrammatical person (95.63%) [*F1*(1, 23) = 5.97, *p* = 0.023; *F2*(1, 253) = 7.63, *p* = 0.006], whereas there were no differences between grammatical number (89.46%) and ungrammatical number (89.85%) [*F1*(1, 23) = 0.06, *p* = 0.81; *F2*(1, 253) = 0.01, *p* = 913]. The analyses by feature factor showed that participants were more accurate with grammatical person (92.45%) than with grammatical number (89.46%) [*F1*(1, 23) = 6.31, *p* = 0.02; *F2*(1, 253) = 5.74, *p* = 0.017], and they were significantly more accurate with ungrammatical person (95.63%) than with ungrammatical number (89.85%) [*F1*(1, 23) = 35.2, *p* < 0.001; *F2*(1, 253) = 23.56, *p* < 0.001].

Finally, a triple TYPE^∗^GRAMMATICALITY^∗^FEATURE was significant in the analysis by subject [*F1*(1, 23) = 8.09, *p* = 0.009; *F2*(1, 253) = 2.77, *p* = 0.097]. The analyses by grammaticality factor showed that in unaccusatives grammatical person condition (94.54%) did not differ from ungrammatical person condition (95.16%) [*F*(1, 23) = 0.19, *p* = 0.667], and neither did grammatical and ungrammatical number (89.98% vs. 90.37%) [*F*(1, 23) = 0.07, *p* = 0.788]. In the unergative conditions participants were significantly more accurate with sentences containing ungrammatical person (96.1%) than with grammatical person (90.37%) [*F*(1, 23) = 13.32, *p* = 0.001], but no differences were found between grammatical (88.93%) and ungrammatical number (89.32%) [*F*(1, 23) = 0.03, *p* = 0.861]. The analyses by type factor revealed participants were more accurate with sentences containing grammatical person feature in unaccusatives (94.54%) than in unergatives (90.37%) [*F*(1, 23) = 12.38, *p* = 0.002], whereas no differences were found between sentences containing ungrammatical person feature in unaccusatives (95.16%) and in unergatives (96.1%) [*F*(1, 23) = 0.66, *p* = 0.423]. With regard to number feature, no differences were found between grammatical unaccusative (89.98%) and (88.93%) unergative predicates, and neither between ungrammatical unaccusative (90.37%) and unergative (89.33%) predicates. Finally, the analyses by feature factor showed that participants were significantly more accurate with grammatical unaccusative sentences containing person feature (94.54%) than with number feature (89.98%) [*F*(1, 23) = 15.89, *p* = 0.001], and similarly ungrammatical unaccusative sentences containing person feature (95.16%) were judged more accurately than number feature (90.37%) [*F*(1, 23) = 13.65, *p* = 0.001]. Regarding unergative predicates, no differences were found between grammatical sentences containing person and number feature [*F*(1, 23) = 0.74, *p* = 0.397], but ungrammatical sentences containing person feature (96.1%) were judged significantly more accurately than ungrammatical sentences containing number feature (89.33%) [*F*(1, 23) = 29.67, *p* < 0.001].

**FIGURE 2:**
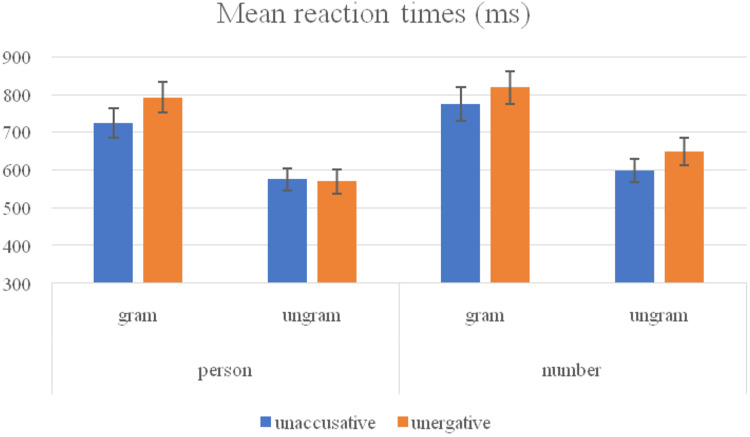
Mean reaction times (ms) and standard deviation error (SDE) of non-native speakers of Basque.

Regarding response times (see Figure 2), the analyses revealed a main TYPE effect [*F1*(1, 23) = 16.41, *p* = 0.001; *F2*(1, 253) = 4.21, *p* = 0.041] indicating participants reacted faster to unaccusative predicates (668.37 ms) than to unergative predicates (707.25 ms). A main GRAMMATICALITY effect [*F1*(1, 23) = 71.51, *p* < 0.001; *F2*(1, 253) = 207.2, *p* < 0.001] revealed that participants were significantly faster reacting to ungrammatical sentences (597.93 ms) compared to their grammatical counterparts (777.68 ms). A FEATURE effect [*F1*(1, 23) = 11.16, *p* = 0.003; *F2*(1, 253) = 9.61, *p* = 0.002] revealed that participants were significantly faster responding to sentences containing person feature (665.47 ms) than number feature (710.15 ms).

### ERP Results

After the baseline correction, epochs with artifacts were rejected, which resulted in the exclusion of approximately 6.91% (*SD* = 2.43) of the trials. Similarly to the procedure reported in Martinez de la Hidalga et al. (2019), 300–400 ms. time window was selected for an early time window and a 400–700 ms. time window was chosen as a late time window.

Regarding the early time window (300–400 ms), the analysis of the lateral electrodes revealed a main GRAMMATICALITY effect [*F*(1, 23) = 18.92, *p* < 0.001] indicating a larger negativity for the ungrammatical conditions as compared to the grammatical ones (1.08 μV vs. 2 μV).

Regarding the midline electrodes, a main effect of GRAMMATICALITY showed that overall ungrammatical conditions (2.04 μV) displayed a larger negativity than grammatical conditions (2.93 μV) [*F*(1, 23) = 11.13, *p* = 0.003]. A significant TYPE^∗^GRAMMATICALITY interaction was found [*F*(1, 23) = 4.9, *p* = 0.037]. Further analysis (by grammaticality) showed no significant differences between ungrammatical (2.36 μV) and grammatical unaccusatives (2.79 μV) [*F*(1, 23) = 2.25, *p* = 0.147] but a larger negativity for the ungrammatical unergative condition (1.73 μV) in comparison to the grammatical unergative condition (3.07 μV) [*F*(1, 23) = 12.67, *p* = 0.002] was found. The comparison by type revealed no differences between the grammatical unaccusative (2.79 μV) and unergative (3.07 μV) conditions [*F*(1, 23) = 0.76, *p* = 0.394], and neither between ungrammatical unaccusative (2.36 μV) and unergative (1.73 μV) conditions [*F*(1, 23) = 1.66, *p* = 0.211].

The analysis of the lateral electrodes in the late time window (400–700 ms) revealed a main GRAMMATICALITY effect [*F*(1, 23) = 60.25, *p* < 0.001] indicating a larger positivity for the ungrammatical conditions as compared to the grammatical ones (2.08 μV vs. −0.03 μV). In addition, a significant main effect of FEATURE emerged [*F*(1, 23) = 13.47, *p* = 0.001], indicating that overall person feature generated a larger positivity as compared to number feature (1.44 μV vs. 0.61 μV).

A significant TYPE^∗^GRAMMATICALITY interaction was found [*F*(1, 23) = 9.34, *p* = 0.006]. Further analysis (by grammaticality) showed a significantly larger positivity for the ungrammatical unaccusative condition (2.32 μV) in comparison to the grammatical one (−0.18 μV) [*F*(1, 23) = 64.23, *p* < 0.001] and a larger positivity for the ungrammatical unergative condition (1.83 μV) in comparison to the grammatical unergative condition (0.12 μV) [*F*(1, 23) = 35.3, *p* < 0.001]. The comparison by type revealed no differences between the grammatical unaccusative (−0.18 μV) and unergative (0.12 μV) conditions [*F*(1, 23) = 1.21, *p* = 0.282] and no differences emerged for ungrammatical unaccusative manipulations (2.32 μV) in comparison to the unergative manipulations (1.83 μV) [*F*(1, 23) = 2.28, *p* = 0.145].

Regarding the midline electrodes, a main effect of GRAMMATICALITY showed that overall ungrammatical conditions (3.59 μV) displayed a larger positivity than grammatical conditions (0.63 μV) [*F*(1, 23) = 59.63, *p* < 0.001]. In addition, a significant FEATURE effect emerged [*F*(1, 23) = 20.94, *p* < 0.001], indicating that overall person feature generated a larger positivity as compared to number feature (2.78 μV vs. 1.44 μV).

**FIGURE 3:**
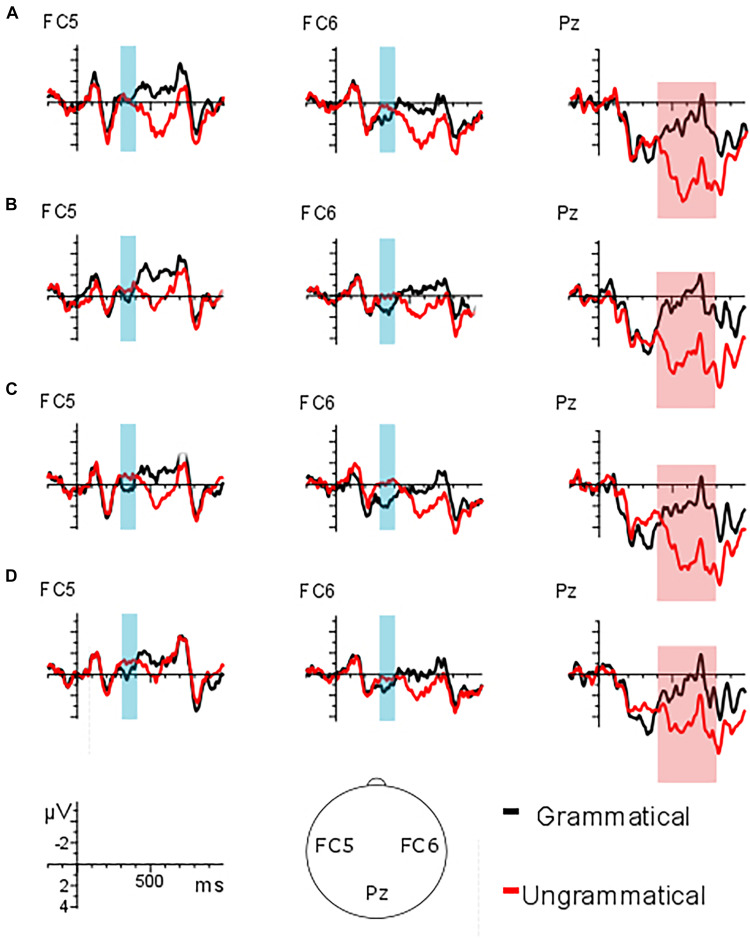
**(A)** Person feature unaccusative predicate condition; **(B)** number feature unaccusative predicate condition; **(C)** person feature unergative predicate condition; **(D)** number feature unergative predicate condition.

**FIGURE 4:**
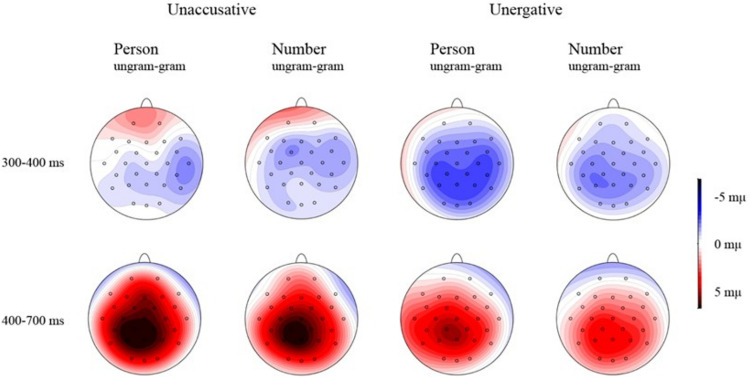
Mean voltage difference maps (grammatical minus ungrammatical).

A significant TYPE^∗^GRAMMATICALITY interaction was found [*F*(1, 23) = 13.45, *p* = 0.001]. Further analysis (by grammaticality) showed a significantly larger positivity for the ungrammatical unaccusative condition (4.03 μV) in comparison to the grammatical one (0.36 μV) (*F*(1, 23) = 56.96, *p* < 0.001) and a larger positivity for the ungrammatical unergative condition (3.14 μV) in comparison to the grammatical number condition (0.9 μV) [*F*(1, 23) = 37.99, *p* < 0.001]. The comparison by type revealed no differences between grammatical unergatives (0.9 μV) and unaccusatives (0.36 μV) [*F*(1, 23) = 3.02, *p* = 0.096], but a slightly larger positivity emerged for ungrammatical unaccusative manipulations (4.03 μV) in comparison to the unergative manipulations (3.14 μV) [*F*(1, 23) = 3.91, *p* = 0.06]. See Figure 3 for the grand average patterns, Figure 4 for the mean voltage difference maps and Table 3 for the summary of the results.

**TABLE 3:** Summary of the ERP results.

		300–400 ms		400–700 ms	
		Lateral	Midline	Lateral	Midline
	*df*	*F*	*F*	*F*	F
GRAM	1.23	***18.92	**11.13	***60.25	***59.63
TYPE	1.23	0.19	0.25	0.11	0.27
FEAT	1.23	0.01	1.26	**13.47	***20.94
TYPE*GRAM	1.23	2.67	4.9*	**9.34	**13.49
FEAT*GRAM	1.23	0.2	0.09	0.88	0.48
TYPE*FEAT*GRAM	1.23	0.83	0.36	0.21	2.04
GRAM*HEM	1.23	2.26	−	3.7	−
TYPE*GRAM*HEM	1.23	0.56	−	0.01	−
FEAT*GRAM*HEM	1.23	^3.2	−	2.97	−
TYPE*FEAT*GRAM*HEM	1.23	0.14	−	1.35	−
GRAM*REGION	2.46	3.29	***14.42	***24.98	***56.36
TYPE*GRAM*REG	2.46	0.49	1.31	0.49	0.66
FEAT*GRAM*REG	2.46	0.3	0.99	0.69	0.29
TYPE*FEAT*GRAM*REG	2.46	0.43	1.2	0.71	0.21
GRAM*HEM*REG	2.46	0.7	−	1.45	−
TYPE*GRAM*HEM*REG	2.46	0.38	−	0.02	−
FEAT*GRAM*HEM*REG	2.46	0.33	−	0.05	−
TYPE*FEAT*GRAM*HEM*REG	2.46	0.14	−	1.13	−

Main effects and interactions with grammaticality are shown. GRAM (grammaticality), TYPE (type), FEAT (feature), HEM (hemisphere), and REG (region). ^p = < 0.1, *p = < 0.05, **p = < 0.01, ***p = < 0.001.

### Native and Non-native Comparison

In order to better understand the similarities and differences between the non-natives and the native speakers tested in Martinez de la Hidalga et al. (2019), we performed an additional analysis comparing both groups directly.

### Behavioral Results

Regarding accuracy, no differences between both groups were found. A marginal main effect of TYPE emerged [*F1*(1, 46) = 2.85, *p* = 0.098; *F2*(1, 252) = 4.18, *p* = 0.041] indicating that overall, both native and non-native participants were more accurate with conditions containing unaccusative predicates (92.76%) compared to unergative predicates (91.82%). The analysis of accuracy revealed a significant main GRAMMATICALITY effect [*F1*(1, 46) = 4.89, *p* = 0.032; *F2*(1, 252) = 13.49, *p* < 0.001] revealing that overall both native and non-native participants were more accurate with conditions containing ungrammatical sentences (93.41%) compared to grammatical sentences (91.16%). The analysis of accuracy also revealed a main FEATURE effect [*F*(1, 46) = 41.51, *p* < 0.001; *F2*(1, 252) = 41.5, *p* < 0.001] suggesting that both natives and non-natives were more accurate with conditions containing person feature (94.17%) compared to conditions containing number feature (90.4%).

A GRAMMATICALITY^∗^FEATURE interaction turned out to be marginally significant in the by subject analysis [*F1*(1, 23) = 3.82, *p* = 0.057; *F2*(1, 252) = 2.4, *p* = 0.118]. The analyses by grammaticality factor showed that participants were significantly less accurate with grammatical person (92.61%) than with ungrammatical person (95.73%) [*F*(1, 46) = 11.57, *p* = 0.001], whereas there were no differences between grammatical number (89.72%) and ungrammatical number (91.08%) [*F*(1, 46) = 1.18, *p* = 0.283]. The analyses by feature factor showed that participants were more accurate with grammatical person (92.61%) than with grammatical number (89.72%) [*F*(1, 46) = 16.23, *p* < 0.001], and they were significantly more accurate with ungrammatical person (95.73%) than with ungrammatical number (91.08%) [*F*(1, 46) = 37.67, *p* < 0.001].

Finally, a triple TYPE^∗^GRAMMATICALITY^∗^FEATURE interaction turned out to be significant [*F1*(1, 46) = 9.28, *p* = 0.004; *F2*(1, 252) = 5.72, *p* = 0.017]. The analyses by grammaticality factor showed that participants were accurate when performing the task with grammatical and ungrammatical unaccusatives containing person feature (94.01% vs. 95.36%) [*F*(1, 46) = 2.32, *p* = 0.134], and similarly with unaccusatives containing number feature (89.72% vs. 91.93%) [*F*(1, 46) = 2.14, *p* = 0.15]. In the unergative conditions participants were significantly more accurate with sentences containing ungrammatical person (96.1%) than with grammatical person (91.21%) [*F*(1, 46) = 15.93, *p* < 0.001], but no differences were found between grammatical (89.72%) and ungrammatical number (90.24%) [*F*(1, 46) = 0.13, *p* = 0.716]. The analyses by type factor revealed that participants were more accurate with sentences containing grammatical person feature in unaccusatives (94.01%) than in unergatives (91.21%) [*F*(1, 46) = 9.41, *p* = 0.004], whereas no differences were found between sentences containing ungrammatical person feature in unaccusatives (95.36%) and in unergatives (96.1%) [*F*(1, 46) = 1.11, *p* = 0.296]. With regard to number feature, no differences were found between grammatical unaccusative (89.72%) and (89.72%) unergative predicates [*F*(1, 46) < 0.01, *p* = 1], and neither between ungrammatical unaccusatives (91.93%) in contrast to ungrammatical unergative (90.24%) predicates [*F*(1, 46) = 2.16, *p* = 0.148]. Finally, the analyses by feature factor showed that participants were significantly more accurate with grammatical unaccusative sentences containing person feature (94.01%) than with number feature (89.72%) [*F*(1, 46) = 22.79, *p* < 0.001], and similarly ungrammatical unaccusative sentences containing person feature (95.36%) were judged more accurately than number feature (91.93%) [*F*(1, 46) = 12.01, *p* = 0.001]. Regarding unergative predicates, no differences were found between grammatical sentences containing person (91.21%) and number feature (89.72%) [*F*(1, 46) = 2.18, *p* = 0.147], but ungrammatical sentences containing person feature (96.1%) were judged significantly more accurately than sentences containing number feature (90.24%) [*F*(1, 46) = 37.89, *p* < 0.001].

The analysis of response times revealed a main TYPE effect [*F1*(1, 46) = 18.21, *p* < 0.001; *F2*(1, 254) = 7.68, *p* < 0.006] indicating that participants reacted faster to unaccusative predicates (637.29 ms) than to unergative predicates (673.08 ms). A main GRAMMATICALITY effect [*F1*(1, 46) = 122.46, *p* < 0.001; *F2*(1, 252) = 453.86, *p* < 0.001] revealed that participants were significantly faster reading ungrammatical sentences (565.16 ms) compared to their grammatical counterparts (745.22 ms). A FEATURE effect [*F1*(1, 46) = 15.48, *p* < 0.001; *F2*(1, 254) = 11.22, *p* = 0.001] revealed that participants were significantly faster reading sentences containing person feature (636.77 ms) than number feature (673.61 ms). A significant TYPE^∗^GRAMMATICALITY interaction emerged [*F1*(1, 46) = 5.04, *p* = 0.03; *F2*(1, 252) = 4.3, *p* = 0.039]. The analyses by grammaticality factor showed that participants reacted faster to ungrammatical unaccusatives (557.11 ms) than to grammatical unaccusative (717.47 ms) predicates [*F1*(1, 46) = 88.49, *p* < 0.001; *F2*(1, 252) = 190.11, *p* < 0.001], and similarly participants responded faster to ungrammatical unergatives (573.21 ms) compared to their grammatical counterparts (772.96 ms) [*F1*(1, 46) = 105.37, *p* < 0.001; *F2*(1, 252) = 274.53, *p* < 0.001]. The analyses by type factor revealed significant differences between grammatical unaccusative and unergative predicates [*F1*(1, 46) = 16.78, *p* < 0.001; *F2*(1, 252) = 0.02, *p* = 0.004], indicating that participants reacted faster to grammatical unaccusatives (717.47 ms) than to grammatical unergatives (772.96 ms), but no differences were found between ungrammatical unaccusatives (557.11 ms) and ungrammatical unergative predicates (573.21 ms) [*F1*(1, 46) = 2.45, *p* = 0.124; *F2*(1, 252) = 0.77, *p* = 0.3.25].

Finally, a triple TYPE^∗^GRAMMATICALITY^∗^FEATURE interaction turned out to be marginally significant in the by subject analysis [*F*(1, 46) = 3.25, *p* = 0.078; *F2*(1, 252) = 3.62, *p* = 0.058]. The analyses by grammaticality factor showed that the unaccusative ungrammatical person condition (550.82 ms) was read faster than the grammatical person condition (705.5 ms) [*F*(1, 46) = 60.54, *p* < 0.001], and similarly for number (563.4 ms vs. 729.44 ms) [*F*(1, 46) = 54.99, *p* < 0.001]. In the unergative conditions participants were significantly faster with sentences containing ungrammatical person (532.75) than with grammatical person (757.99 ms) [*F*(1, 46) = 109.43, *p* < 0.001], and similarly they were faster with ungrammatical number (613.67 ms) than with grammatical number (787.93 ms) [*F*(1, 46) = 59.2, *p* < 0.001]. The analyses by type factor revealed participants were faster with sentences containing grammatical person feature in unaccusatives (705.5 ms) than in unergatives (757.99 ms) [*F*(1, 46) = 8.4, *p* = 0.006], whereas no differences emerged between ungrammatical person feature in unaccusatives (550.82 ms) and unergatives (532.75 ms) [*F*(1, 46) = 1.23, *p* = 0.273]. With regard to number feature, participants responded faster to grammatical sentences in unaccusatives (729.44 ms) than in unergatives (787.93 ms) [*F*(1, 46) = 10.73, *p* = 0.002], and similarly participants reacted faster to ungrammatical unaccusative (563.4 ms) than to ungrammatical unergatives (613.67 ms) [*F*(1, 46) = 10.45, *p* = 0.002]. Finally, the analyses by feature factor showed that participants reacted similarly to grammatical unaccusative sentences containing person feature (705.76 ms) and number feature (729.35) [*F*(1, 46) = 1.62, *p* = 0.21], and similarly there were no differences between ungrammatical unaccusative sentences containing person feature (550.81 ms) and number feature (561.63 ms) [*F*(1, 46) = 0.75, *p* = 0.391]. Regarding unergative predicates, no differences were found between grammatical sentences containing person (758.07 ms) and number feature (787.54 ms) [*F*(1, 46) = 2.62, *p* = 0.113], but ungrammatical sentences containing person feature (533.36 ms) were judged significantly faster than sentences containing number feature (612.96 ms) [*F*(1, 46) = 25.48, *p* < 0.001]. Overall, no differences between groups were observed in the behavioral measures.

### ERP Results

Regarding the early time window (300–400 ms), the analysis of the lateral electrodes revealed a main GRAMMATICALITY effect [*F*(1, 46) = 51.43, *p* < 0.001] indicating a larger negativity for the ungrammatical conditions as compared to the grammatical ones (1 μV vs. 2.13 μV).

A significant FEATURE^∗^GRAMMATICALITY interaction was found as well [*F*(1, 46) = 4.5, *p* = 0.039]. Further analysis (by grammaticality) showed a significantly larger negativity for the ungrammatical person condition (0.75 μV) in comparison to the grammatical one (2.15 μV) [*F*(1, 46) = 42.5, *p* < 0.001] and a larger negativity for the ungrammatical number condition (1.24 μV) in comparison to the grammatical number condition (2.11 μV) [*F*(1, 46) = 20.38, *p* < 0.001]. The comparison by feature revealed no differences between the grammatical person (2.15 μV) and number feature (2.11 μV) conditions [*F*(1, 47) = 0.04, *p* = 0.841], but it revealed a larger negativity for the ungrammatical person manipulations (0.75 μV) in comparison to the number manipulations (1.24 μV) [*F*(1, 46) = 6.51, *p* = 0.014].

Regarding the midline electrodes, a main effect of GRAMMATICALITY showed that overall ungrammatical conditions (2.35 μV) displayed a larger negativity than grammatical conditions (3.29 μV) [*F*(1, 46) = 20.43, *p* > 0.001].

A significant TYPE^∗^GRAM^∗^GROUP interaction [*F*(1, 46) = 6.4, *p* = 0.015] showed (by grammaticality factor) that natives revealed a larger negativity for the ungrammatical unaccusative condition (2.46 μV) than for the grammatical unaccusative condition (3.66 μV) [*F*(1, 46) = 13.5, *p* = 0.001] and also a larger negativity for the ungrammatical unergative condition (2.84 μV) than for the grammatical unergative condition (3.62 μV) [*F*(1, 46) = 4.48, *p* = 0.040], whereas non-natives only elicited a larger negativity for the ungrammatical unergative condition (1.7 μV) compared to the grammatical unergative condition (3.07 μV) [unaccusative: *F*(1, 46) = 1.76, *p* = 0.191; unergative: *F*(1, 46) = 13.36, *p* = 0.001]. In the analysis by type factor, no differences were found between grammatical unaccusative and unergative conditions neither in natives [*F*(1, 46) = 0.2, *p* = 0.889] nor in non-natives [*F*(1, 46) = 0.71, *p* = 0.405], and similarly no differences were found between ungrammatical unaccusative and unergative conditions neither in natives [*F*(1, 46) = 0.95, *p* = 0.334] nor in non-natives [*F*(1, 46) = 2.67, *p* = 0.109]. The *T*-test showed that the negativity elicited in non-natives was marginally larger than in natives for the unergative ungrammatical condition (1.7 μV vs. 2.84 μV) [*F*(1, 46) = 0.45, *p* = 0.09].

Regarding the 400–700 ms time window, the analysis of the lateral electrodes revealed a main GRAMMATICALITY effect [*F*(1, 46) = 103.8, *p* < 0.001] indicating a larger positivity for the ungrammatical conditions as compared to the grammatical ones (2.33 μV vs. 0.08 μV). A significant main effect of FEATURE also emerged [*F*(1, 46) = 13.14, *p* = 0.001], indicating that overall person feature generated a larger positivity as compared to number feature (1.48 μV vs. 0.93 μV).

A significant TYPE^∗^GRAMMATICALITY interaction was found [*F*(1, 46) = 6.1, *p* = 0.017]. Further analysis (by grammaticality) showed a significantly larger positivity for the ungrammatical unaccusative condition (2.54 μV) in comparison to the grammatical one (0.02 μV) [*F*(1, 46) = 108.41, *p* < 0.001] and a larger positivity for the ungrammatical unergative condition (2.13 μV) in comparison to the grammatical number condition (0.13 μV) [*F*(1, 46) = 65.39, *p* < 0.001]. The comparison by type revealed no differences between the grammatical unaccusative (0.02 μV) and unergative (0.13 μV) conditions [*F*(1, 46) = 0.4, *p* = 0.529], but a larger positivity emerged for ungrammatical unaccusative manipulations (2.54 μV) in comparison to the ungrammatical unergative manipulations (2.13 μV) [*F*(1, 46) = 4.52, *p* = 0.039]. A significant TYPE^∗^GRAM^∗^REGION [*F*(2, 46) = 5.82, *p* = 0.012] interaction also showed a larger positivity for ungrammatical conditions in comparison to grammatical conditions in unaccusatives [frontal: *F*(1, 46) = 31.78, *p* < 0.001; central: *F*(1, 46) = 127.11, *p* < 0.001; posterior: *F*(1, 46) = 123.52, *p* < 0.001] and in unergatives [frontal: *F*(1, 46) = 27.37, *p* < 0.001; central: *F*(1, 46) = 76.9, *p* < 0.001; posterior: *F*(1, 46) = 54.44, *p* < 0.001] in all three regions. The comparison by type revealed no differences between grammatical conditions, but it revealed significant differences between ungrammatical unaccusative and ungrammatical unergative conditions in central [*F*(1, 46) = 5.31, *p* = 0.026] and posterior [*F*(1, 46) = 5.2, *p* = 0.027] electrodes, thus indicating that ungrammatical unaccusatives elicit a larger positivity than ungrammatical unergatives.

Regarding the midline electrodes, a main effect of FEATURE [*F*(1, 46) = 21.85, *p* < 0.001] showed that overall person (3.01 μV) displayed a larger positivity than number (2.15 μV). A further main effect of GRAMMATICALITY [*F*(1, 46) = 120.16, *p* > 0.001] revealed that overall ungrammatical conditions (4.28 μV) displayed a larger positivity than grammatical conditions (0.87 μV).

A FEATURE^∗^GROUP interaction [*F*(1, 46) = 6.88, *p* = 0.012] emerged. The analysis by feature factor revealed no differences between person and number regarding natives [*F*(1, 46) = 2.1, *p* = 0.154], but it showed that person elicited a larger positivity than number in non-natives (2.78 μV vs. 1.44 μV) [*F*(1, 46) = 26.63, *p* < 0.001]. The *t*-test showed that there were no group differences regarding person feature (3.23 μV vs. 2.78 μV) [*F*(1, 46) = 0.46, *p* = 0.505], but natives elicited a larger positivity than non-natives with regard to number feature (2.86 μV vs. 1.44 μV) [*F*(1, 46) = 0.02, *p* = 0.022].

A TYPE^∗^GRAM interaction was also found [*F*(1, 46) = 9.19, *p* = 0.004]. Further analysis by grammaticality showed that ungrammatical unaccusatives (4.64 μV) elicited a larger positivity than grammatical unaccusatives (0.8 μV) [*F*(1, 46) = 112.21, *p* > 0.001], and similarly ungrammatical unergatives elicited a larger positivity (3.92 μV) than grammatical unergatives (0.94 μV) [*F*(1, 46) = 81.51, *p* > 0.001]. Analysis by type showed that there are no differences between grammatical unaccusatives (0.8 μV) and grammatical unergatives (0.94 μV) [*F*(1, 46) = 0.36, *p* = 0.55], whereas ungrammatical unaccusatives (4.64 μV) elicited a larger positivity than ungrammatical unergatives (3.92 μV) [*F*(1, 46) = 7.5, *p* = 0.009].

A marginally significant TYPE^∗^GRAM^∗^GROUP interaction [*F*(1, 46) = 4.02, *p* = 0.051] showed (by grammaticality factor) that both natives and non-natives revealed a larger positivity for the ungrammatical unaccusative condition than for the grammatical unaccusative condition [natives: *F*(1, 46) = 60.29, *p* < 0.001; non-natives: *F*(1, 46) = 50.3, *p* < 0.001] and also a larger positivity for the ungrammatical unergative condition than for the grammatical unergative condition [natives: *F*(1, 46) = 69.71, *p* < 0.001; non-natives: *F*(1, 46) = 25.21, *p* < 0.001]. In the analysis by type factor, no differences were found between the grammatical unaccusative and unergative conditions neither in natives [*F*(1, 46) = 0.62, *p* = 0.435] nor in non-natives [*F*(1, 46) = 2.75, *p* = 0.104], and no differences were found between ungrammatical unaccusative and unergative conditions in natives either [*F*(1, 46) = 2.16, *p* = 0.149]. However, in non-natives ungrammatical unaccusatives elicited a larger positivity than ungrammatical unergatives (4.02 μV vs. 3.14 μV) [*F*(1, 46) = 5.67, *p* = 0.021]. The *t*-test revealed that natives elicited a marginally larger positivity than non-natives in the unergative ungrammatical condition (4.71 μV vs. 3.14 μV) [*F*(1, 46) = 1.98, *p* = 0.054].

There was also a significant TYPE^∗^FEATURE^∗^GRAM interaction [*F*(2, 46) = 7.22, *p* = 0.01]. The analysis by grammaticality factor showed that the positivity elicited by the ungrammatical sentences was significantly larger than that yielded by the grammatical sentences in all the conditions [unaccusative person: *F*(1, 23) = 75.1, *p* < 0.001; unaccusative number: *F*(1, 23) = 100.22, *p* < 0.001; unergative person: *F*(1, 23) = 93.01, *p* < 0.001; unergative number: *F*(1, 23) = 36.42, *p* < 0.001]. The analysis by type factor revealed no differences across predicate type regarding person feature [grammatical person: *F*(1, 23) = 1.11, *p* < 0.298; ungrammatical person: *F*(1, 23) = 0.05, *p* = 0.828]. Nevertheless, regarding number feature grammatical unergatives elicited a larger positivity than grammatical unaccusatives (0.83 μV vs. 0.22 μV) [grammatical number: *F*(1, 23) = 5.81, *p* = 0.02], and ungrammatical unaccusatives elicited a larger positivity than ungrammatical unergatives (4.24 μV vs. 3.29 μV) [*F*(1, 23) = 8.36, *p* = 0.006]. Concurrently, the analysis by feature factor showed that person elicited a larger positivity than number feature in the grammatical (1.38 μV vs. 0.22 μV) and ungrammatical (5.05 μV vs. 4.24 μV) unaccusative condition [grammatical: *F*(1, 23) = 15.07, *p* < 0.001; ungrammatical: *F*(1, 23) = 4.92, *p* = 0.031]. Regarding unergatives, no differences were found between person and number in the grammatical condition [*F*(1, 23) = 0.34, *p* = 0.563], but a significant difference was found between person and number in the ungrammatical condition, showing that both natives and non-natives generate a larger positivity when processing ungrammatical person unergatives (4.55μV) than ungrammatical number unergatives (3.3μV) [*F*(1, 23) = 31.32, *p* < 0.001].

Finally, a marginally significant GRAM^∗^REGION^∗^GROUP interaction also emerged [*F*(2, 46) = 3.19, *p* = 0.056]. The analysis by grammaticality showed that both natives and non-natives elicited a larger positivity in ungrammatical sentences than in their grammatical counterparts in frontal electrodes [natives: *F*(1, 46) = 34.78, *p* > 0.001; non-natives: *F*(1, 46) = 25.49, *p* > 0.001], central electrodes [natives: *F*(1, 46) = 70.04, *p* > 0.001; non-natives: *F*(1, 46) = 39.1, *p* > 0.001], and posterior electrodes [natives: *F*(1, 47) = 94.26, *p* > 0.001; non-natives: *F*(1, 46) = 51.57, *p* > 0.001]. The analysis by group showed that no differences obtained between native and non-natives’ grammatical sentences in frontal electrodes [*F*(1, 46) = 0.55, *p* = 0.284], nor central electrodes [*F*(1, 46) = 0.35, *p* = 0.523], nor posterior electrodes [*F*(1, 46) = 0.14, *p* = 0.484]. Similarly, no differences between native and non-natives obtained in ungrammatical sentences in frontal electrodes [*F*(1, 46) = 0.44, *p* = 0.177] and in central electrodes [*F*(1, 46) = 0.72, *p* = 0.114], but natives elicited a marginally larger positivity in posterior electrodes compared to non-natives (6.82 μV vs. 4.97 μV) [*F*(1, 46) = 2.19, *p* = 0.065] (See Table 4 for the summary of the results).

**TABLE 4:** Summary of the ERP results.

		300–400 ms		400–700 ms	
		Lateral	Midline	Lateral	Midline
	*df*	*F*	*F*	*F*	*F*
GROUP	1.23	0.01	1.42	0.81	2.36
GRAM	1.23	***51.43	***20.43	***103.8	***120.61
GRAM*GROUP	1.23	1.79	0.06	0.47	2.18
TYPE	1.23	0.0	0.0	0.86	2.02
TYPE*GROUP	1.23	0.62	0.64	1.3	0.32
FEAT	1.23	2.65	0.25	**13.14	***21.85
FEAT*GROUP	1.23	^3.07	^3.96	^3.55	*6.88
TYPE*GRAM	1.23	0.69	0.84	**6.1	**9.19
TYPE*GRAM*GROUP	1.23	2.26	*6.4	1.62	^4.02
FEAT*GRAM	1.23	*4.5	2.21	0.06	1.17
FEAT*GRAM*GROUP	1.23	1.87	0.97	0.96	0.01
TYPE*FEAT*GRAM	1.23	2.53	1.84	2.47	*7.22
TYPE*FEAT*GRAM*GROUP	1.23	0.11	0.3	1.07	0.79
GRAM*HEM	1.23	0.66	−	1.58	−
GRAM*HEM*GROUP	1.23	1.11	−	1.35	−
TYPE*GRAM*HEM	1.23	0.83	−	0.74	−
TYPE*GRAM*HEM*GROUP	1.23	^3.41	−	0.96	−
FEAT*GRAM*HEM	1.23	0.03	−	0.11	−
FEAT*GRAM*HEM*GROUP	1.23	^2.94	−	^3.26	−
TYPE*FEAT*GRAM*HEM	1.23	0.32	−	^2.93	−
TYPE*FEAT*GRAM*HEM*GROUP	1.23	0.0	−	0.0	−
GRAM*REGION	2.46	**10.82	***13.98	***41.46	***77.1
GRAM*REGION*GROUP	2.46	3.13	0.48	0.34	^3.19
TYPE*GRAM*REG	2.46	3.03	1.28	*5.82	0.91
TYPE*GRAM*REG*GROUP	2.46	0.82	0.08	2.62	0.83
FEAT*GRAM*REG	2.46	1.1	^2.88	1.62	0.53
FEAT*GRAM*REG*GROUP	2.46	0.14	0.43	0.1	0.37
TYPE*FEAT*GRAM*REG	2.46	0.3	2.05	1.38	2.4
TYPE*FEAT*GRAM*REG*GROUP	2.46	0.22	0.02	0.26	1.02
GRAM*HEM*REG	2.46	*5.15	−	0.7	−
GRAM*HEM*REG*GROUP	2.46	1.84	−	0.53	−
TYPE*GRAM*HEM*REG	2.46	0.01	−	0.19	−
TYPE*GRAM*HEM*REG*GROUP	2.46	0.52	−	0.15	−
FEAT*GRAM*HEM*REG	2.46	0.27	−	0.3	−
FEAT*GRAM*HEM*REG*GROUP	2.46	0.23	−	0.62	−
TYPE*FEAT*GRAM*HEM*REG	2.46	0.03	−	0.73	−
TYPE*FEAT*GRAM*HEM*REG*G	2.43	0.28	−	0.31	−

Main effects and interactions with grammaticality are shown. GRAM (grammaticality), TYPE (type), FEAT (feature), HEM (hemisphere) and REG (region). ^p = < 0.1, *p = < 0.05, **p = < 0.01, ***p = < 0.001.

## Discussion

The present study aimed to examine whether native-like processing can be achieved in a second language when the linguistic features tested are shared by L1 and L2. To this purpose we tested early and high proficient non-native speakers of Basque while processing intransitive predicates (unergatives/unaccusatives) and phi-features (person/number). These results were compared to those previously obtained from native speakers in the same tasks (Martinez de la Hidalga et al., 2019).

Overall, these early and proficient non-native speakers were indistinguishable from natives: (a) in the acceptability task, non-natives were faster and more accurate in the unaccusative condition and in the person violation condition; (b) they displayed a larger positivity for unaccusative violations than for unergative violations; (c) in the unergative condition, they displayed a larger positivity for person than for number feature violations; (d) number violations elicited larger positivity in the unaccusative condition than in the unergative condition.

In Martinez de la Hidalga et al. (2019), we found differences in the processing of unaccusative and unergative predicates, thus supporting the Unaccusative Hypothesis. Nevertheless, we showed that in Basque, contrary to the predictions made by the Unaccusative Hypothesis, unaccusative predicates are not costlier to process than unergative predicates. We thus provided new evidence in support of the view which advocates for no syntactic movement for subjects of unaccusatives in Basque (Laka, 2006a, b; Levin, 1983). Evidence for greater processing costs for unaccusative predicates compared to unergative predicates have been repeatedly found in nominative-accusative languages (Bastiaanse and van Zonneveld, 2005; Friedmann et al., 2008; Koring et al., 2012; Meltzer-Asscher et al., 2015; Dekydtspotter and Seo, 2017; *inter alia*). One possibility for Spanish bilinguals may have been to show traces of the processing of their native language (nominative-accusative) when processing intransitive predicates in Basque (ergative-absolutive), where no extra processing costs are found for unaccusative predicates. Instead, early and high proficient Spanish-Basque bilinguals processed intransitive predicates as do natives, and displayed measures of greater processing costs for unergatives than for unaccusatives.

Although by and large L2 speakers behaved native-like, their electrophysiological activity revealed a few minor differences: (a) non-natives did not generate a N400 in response to unaccusative violations; (b) the P600 generated by violations in the unergative condition was smaller than that generated by natives; (c) regarding phi-features, non-natives generated smaller negativity for number than natives.

Regarding (a) a lack of negativity in non-natives has been often reported in the literature (Hagoort et al., 1993; Osterhout et al., 1996; Münte et al., 1997; Hagoort and Brown, 2000; Alemán-Bañón and Rothman, 2019; *i.a*.). According to Hahne (2001) smaller or absent negativity in non-native speakers when detecting ungrammaticality may be due to a reduced degree of automaticity in the activation of processing resources.

Regarding (b) quantitative differences in the P600 have also been reported for non-native speakers and are usually attributed to differences in the frequency of use (Osterhout et al., 2006; Rossi et al., 2006). We cannot discard the possibility that differences in the frequency of use had an effect in the processing of intransitive predicate. This factor shall be taken into consideration in future studies in order to discard this possibility.

Besides, there is a morphological difference between the native and non-native language: the processing of unergative subject-verb agreement in Basque involves the processing of ergative case, a case marker not existing in Spanish. A speculative explanation for the slight enhancement of the N400 for unergatives and the decrease of the P600 for non-natives could be that non-natives displayed a smaller sensitivity for processing a case marking not present in their native language, compared to unaccusatives, where case is morphologically unmarked.

Finally, regarding (c) phi features, non-natives generated smaller negativity for number than natives. In any case, the effect and tendency to generate larger negativity for person than for number was the same for native and non-native speakers.

To conclude, in the present study we provided evidence that native-like processing is attainable for early and proficient bilinguals whenever the linguistic properties are shared by their two languages, as suggested by the LDH. We, therefore, argue that cross-linguistic similarity is an important factor that deserves further consideration to better understand what drives bilinguals to process a second language *native-like*.

## Data Availability Statement

The raw data supporting the conclusions of this article will be made available by the authors, without undue reservation.

## Ethics Statement

The studies involving human participants were reviewed and approved by the Ethics Committee for Research involving human beings at the University of the Basque Country (UPV/EHU). The patients/participants provided their written informed consent to participate in this study.

## Author Contributions

GM: data acquisition, analysis and writing. AZ: conceptualization, experimental design, assistance with data analysis, and writing. IL: conceptualization, experimental design and writing. All authors contributed to the article and approved the submitted version.

## Conflict of Interest

The authors declare that the research was conducted in the absence of any commercial or financial relationships that could be construed as a potential conflict of interest.

## Publisher’s Note

All claims expressed in this article are solely those of the authors and do not necessarily represent those of their affiliated organizations, or those of the publisher, the editors and the reviewers. Any product that may be evaluated in this article, or claim that may be made by its manufacturer, is not guaranteed or endorsed by the publisher.

## Footnotes

1 One participant had Catalan as mother tongue; given the typological similarity between Catalan and Spanish, those data were not discarded from the final analysis.

## References

[B1] Alemán-Bañón J. Rothman J. (2019). Being a participant matters: event-related potentials show that markedness modulates person agreement in Spanish. *Front. Psychol.* 10:746. 10.3389/fpsyg.2019.00746 31068847 PMC6491576

[B2] Bastiaanse R. van Zonneveld R. (2005). Sentence production with verbs of alternating transitivity in agrammatic Broca’s aphasia. *J. Neurolinguist.* 18 57–66. 10.1016/j.jneuroling.2004.11.006

[B3] Brice H. Mencl W. E. Frost S. J. Bick A. S. Rueckl J. G. Pugh K. R. (2019). Neurobiological signatures of L2 proficiency: evidence from a bi-directional cross-linguistic study. *J. Neurolinguist.* 50 7–16. 10.1016/j.jneuroling.2018.02.004 PMC6452641 30976136

[B4] Burzio L. (1986). *Italian Syntax: A Government-Binding Approach.* Dordrecht: Springer.

[B5] Caffarra S. Molinaro N. Davidson D. Carreiras M. (2015). Second language syntactic processing revealed through event-related potentials: an empirical review. *Neurosci. Biobehav. Rev.* 51C 31–47. 10.1016/j.neubiorev.2015.01.010 25614131

[B6] Carminati M. N. (2005). Processing reflexes of the feature hierarchy (Person > Number > Gender) and implications for linguistic theory. *Lingua* 115, 259–285. 10.1016/j.lingua.2003.10.006

[B100] Clahsen H. Felser C. (2006). Grammatical processing in language learners. *Appl. Psychol.* 27, 3–42. 10.1017/S0142716406060024 19337839

[B7] Dekydtspotter L. Seo H.-K. (2017). Transitivity in the processing of intransitive clauses: a category-based prediction in low-intermediate learners of english. *Stud. Sec. Lang. Acquisit.* 39 527–552. 10.1017/s0272263116000188

[B8] Díaz B. Erdocia K. de Menezes R. F. Mueller J. L. Sebastián-Gallés N. Laka I. (2016). Electrophysiological correlates of second-language syntactic processes are related to native and second language distance regardless of age of acquisition. *Front. Psychol.* 7:133. 10.3389/fpsyg.2016.00133 26903930 PMC4751279

[B9] Díaz B. Sebastián-Gallés N. Erdocia K. Mueller J. L. Laka I. (2011). On the cross-linguistic validity of electrophysiological correlates of morphosyntactic processing: a study of case and agreement violations in Basque. *J. Neurolinguist.* 24 357–373. 10.1016/j.jneuroling.2010.12.003

[B10] Erdocia K. Zawiszewski A. Laka I. (2014). Word order processing in a second language: from VO to OV. *J. Psycholinguist. Res.* 43 815–837. 10.1007/s10936-013-9280-4 24368710

[B11] Foucart A. Frenck-Mestre C. (2012). Can late L2 learners acquire new grammatical features? Evidence from ERPs and eye-tracking. *J. Mem. Lang.* 66 226–248. 10.1016/j.jml.2011.07.007

[B12] Friederici A. D. Steinhauer K. Pfeifer E. (2002). Brain signatures of artificial language processing: evidence challenging the critical period hypothesis. *Proc. Natl. Acad. Sci. U.S.A.* 99 529–534. 10.1073/pnas.012611199 11773629 PMC117594

[B13] Friedmann N. Taranto G. Shapiro L. P. Swinney D. (2008). The leaf fell (the leaf): the online processing of unaccusatives. *Linguist. Inq.* 39 355–377. 10.1162/ling.2008.39.3.355 22822348 PMC3399662

[B14] Gratton G. Coles M. G. Donchin E. (1983). A new method for off-line removal of ocular artifact. *Electroencephalogr. Clin. Neurophysiol.* 55 468–484. 10.1016/0013-4694(83)90135-9 6187540

[B15] Greenhouse S. W. Geisser S. (1959). On methods in the analysis of profile data. *Psychometrika* 24 95–112. 10.1007/BF02289823

[B16] Hagoort P. Brown C. Groothusen J. (1993). The syntactic positive shift (sps) as an ERP measure of syntactic processing. *Lang. Cogn. Process.* 8 439–483. 10.1080/01690969308407585

[B17] Hagoort P. Brown C. M. (2000). ERP effects of listening to speech compared to reading: the P600/SPS to syntactic violations in spoken sentences and rapid serial visual presentation. *Neuropsychologia* 38 1531–1549. 10.1016/S0028-3932(00)00053-1 10906378

[B18] Hahne A. (2001). What’s different in second-language processing? Evidence from event-related brain potentials. *J. Psycholinguist. Res.* 30 251–266. 11523274 10.1023/a:1010490917575

[B19] Hartshorne J. K. Tenenbaum J. B. Pinker S. (2018). A critical period for second language acquisition: evidence from 2/3 million english speakers. *Cognition* 177 263–277. 10.1016/j.cognition.2018.04.007 29729947 PMC6559801

[B20] Hartsuiker R. J. Pickering M. J. Veltkamp E. (2004). Is syntax separate or shared between languages? *Psychol. Sci.* 15 409–414. 10.1111/j.0956-7976.2004.00693.x 15147495

[B21] Hernandez A. E. Hofmann J. Kotz S. A. (2007). Age of acquisition modulates neural activity for both regular and irregular syntactic functions. *NeuroImage* 36 912–923. 17490895 10.1016/j.neuroimage.2007.02.055 PMC1995424

[B22] Hernandez A. Li P. MacWhinney B. (2005). The emergence of competing modules in bilingualism. *Trends Cogn. Sci.* 9, 220–225. 10.1016/j.tics.2005.03.003 15866148 PMC4107303

[B23] Koring L. Mak P. Reuland E. (2012). The time course of argument reactivation revealed: using the visual world paradigm. *Cognition* 123 361–379. 10.1016/j.cognition.2012.02.011 22475295

[B24] Kotz S. A. (2009). A critical review of ERP and fMRI evidence on L2 syntactic processing. *Brain Lang.* 109 68–74. 18657314 10.1016/j.bandl.2008.06.002

[B25] Kotz S. A. Holcomb P. J. Osterhout L. (2008). ERPs reveal comparable syntactic sentence processing in early bilinguals and monolinguals. *Acta Psychol.* 128 514–527. 10.1016/j.actpsy.2007.10.003 18061129 PMC2711869

[B26] Laka I. (2006a). Deriving split ergativity in the progressive. *Ergativity* 65 173–195.

[B27] Laka I. (2006b). “On the nature of case in Basque: structural or inherent?,” in *Organizing Grammar*, eds Broekhuis H. Corver N. Huijbregts R. Kleinhenz U. Koster J. (Berlin: Walter de Gruyter).

[B28] Lenneberg E. H. (1967). The biological foundations of language. *Hosp. Pract.* 2, 59–67.

[B29] Levin B. (1983). *On the Nature of Ergativity.* [Doctoral dissertation]. Cambridge, MA: Massachussets Institute of Technology.

[B30] MacWhinney B. (2005). Extending the competition model. *Int. J. Bilingualism* 9 69–84. 10.1177/13670069050090010501

[B31] Mancini S. (2018). *Features and Processing in Agreement.* Cambridge, MA: Cambridge Scholars Publishing.

[B32] Mancini S. Molinaro N. Rizzi L. Carreiras M. (2011). A person is not a number: discourse involvement in subject–verb agreement computation. *Brain Res.* 1410 64–76. 10.1016/j.brainres.2011.06.055 21798519

[B33] Martinez de la Hidalga G. Zawiszewski A. Laka I. (2019). Eppur non si muove: experimental evidence for the Unaccusative Hypothesis and distinct Φ-feature processing in Basque. *Glossa* 4:120. 10.5334/gjgl.829

[B34] Matzke M. Mai H. Nager W. Rüsseler J. Münte T. (2002). The costs of freedom: an ERP – study of non-canonical sentences. *Clin. Neurophysiol.* 113 844–852. 10.1016/s1388-2457(02)00059-7 12048043

[B35] Meltzer-Asscher A. Mack J. E. Barbieri E. Thompson C. K. (2015). How the brain processes different dimensions of argument structure complexity: evidence from fMRI. *Brain Lang.* 142 65–75. 10.1016/j.bandl.2014.12.005 25658635 PMC4336802

[B36] Montrul S. (2005). On knowledge and development of unaccusativity in Spanish L2 acquisition. *Linguistics* 43 1153–1190.

[B37] Münte T. F. Matzke M. Johannes S. (1997). Brain activity associated with syntactic incongruencies in words and pseudo-words. *J. Cogn. Neurosci.* 9 318–329. 10.1162/jocn.1997.9.3.318 23965010

[B38] Ojima S. Nakata H. Kakigi R. (2005). An ERP study of second language learning after childhood: effects of proficiency. *J. Cogn. Neurosci.* 17 1212–1228. 16197679 10.1162/0898929055002436

[B39] Ortiz de Urbina J. (1989). *Parameters in the Grammar of Basque: A GB Approach to Basque Syntax.* Dordrecht: Foris.

[B40] Oshita H. (2001). The unaccusative trap in second language acquisition. *Stud. Sec. Lang. Acquisit.* 23 279–304. 10.1017/s0272263101002078

[B41] Osterhout L. McKinnon R. Bersick M. Corey V. (1996). On the language specificity of the brain response to syntactic anomalies: is the syntactic positive shift a member of the p300 family? *J. Cogn. Neurosci.* 8 507–526. 10.1162/jocn.1996.8.6.507 23961982

[B42] Osterhout L. McLaughlin J. Pitkänen I. Frenck-Mestre C. Molinaro N. (2006). Novice learners, longitudinal designs, and event-related potentials: a means for exploring the neurocognition of second language processing. *Lang. Learn.* 56 199–230.

[B43] Perlmutter D. M. (1978). Impersonal passives and the unaccusative hypothesis. *Annu. Meet. Berkeley Linguist. Soc.* 4 157–190. 10.3765/bls.v4i0.2198 26464298

[B44] Rossi S. Gugler M. F. Friederici A. D. Hahne A. (2006). The impact of proficiency on syntactic second-language processing of German and Italian: evidence from event-related potentials. *J. Cogn. Neurosci.* 18 2030–2048. 10.1162/jocn.2006.18.12.2030 17129189

[B45] Schwartz B. Sprouse R. (1996). L2 cognitive states and the full transfer/full access model. *Sec. Lang. Res.* 12 40–72.

[B46] Steinhauer K. White E. J. Drury J. E. (2009). Temporal dynamics of late second language acquisition: evidence from event-related brain potentials. *Second Lang. Res.* 25, 13–41. 10.1177/0267658308098995

[B47] Tsimpli I. M. Dimitrakopoulou M. (2007). The interpretability hypothesis: Evidence from wh-interrogatives in second language acquisition. *Second Lang. Res.* 23, 215–242. 10.1177/0267658307076546

[B48] Vandenberghe B. Perez M. M. Reynvoet B. Desmet P. (2019). The role of Event-Related Potentials (ERPs) as sensitive measures in L2 vocabulary acquisition research. *J. Eur. Sec. Lang. Assoc.* 3 35–45. 10.22599/jesla.60 33001059

[B49] Wartenburger I. Heekeren H. R. Abutalebi J. Cappa S. F. Villringer A. Perani D. (2003). Early setting of grammatical processing in the bilingual brain. *Neuron* 37 159–170. 12526781 10.1016/s0896-6273(02)01150-9

[B50] Weber-Fox C. M. Neville H. J. (1996). Maturational constraints on functional specializations for language processing: ERP and behavioral evidence in bilingual speakers. *J. Cogn. Neurosci.* 8 231–256. 10.1162/jocn.1996.8.3.231 23968150

[B51] Yuan B. (1999). Acquiring the unaccusative unergative distinction in a second language: evidence from English-speaking learners of L2 Chinese. *Linguistics* 37 275–296.

[B52] Zawiszewski A. Gutiérrez E. Fernández B. Laka I. (2011). Language distance and non-native syntactic processing: evidence from event-related potentials. *Bilingualism Lang. Cogn.* 14 400–411. 10.1017/s1366728910000350

[B53] Zawiszewski A. Laka I. (2020). Bilinguals processing noun morphology: evidence for the language distance hypothesis from event-related potentials. *J. Neurolinguist.* 55:100908. 10.1016/j.jneuroling.2020.100908

[B54] Zawiszewski A. Santesteban M. Laka I. (2016). Phi-features reloaded: an event-related potential study on person and number agreement processing. *Appl. Psycholinguist.* 37 601–626. 10.1017/s014271641500017x

